# EBI2 Is Temporarily Upregulated in MO3.13 Oligodendrocytes during Maturation and Regulates Remyelination in the Organotypic Cerebellar Slice Model

**DOI:** 10.3390/ijms22094342

**Published:** 2021-04-21

**Authors:** Maria Velasco-Estevez, Nina Koch, Ilona Klejbor, Stephane Laurent, Kumlesh K. Dev, Andrzej Szutowicz, Andreas W. Sailer, Aleksandra Rutkowska

**Affiliations:** 1 Department of Laboratory Medicine, Medical University of Gdańsk, 80-210 Gdańsk, Poland; mvelasco@tcd.ie (M.V.-E.); kochn@tcd.ie (N.K.); andrzej.szutowicz@gumed.edu.pl (A.S.); 2 Department of Anatomy and Neurobiology, Medical University of Gdańsk, 80-210 Gdańsk, Poland; ilona.klejbor@gumed.edu.pl; 3 Chemical Biology and Therapeutics/Disease Area X/Liver, Novartis Institutes for BioMedical Research, Novartis Pharma AG, CH-4056 Basel, Switzerland; stephane.laurent@novartis.com (S.L.); andreas.sailer@novartis.com (A.W.S.); 4 School of Medicine, Trinity College Dublin, Dublin 2, Ireland; devk@tcd.ie

**Keywords:** EBI2 receptor, remyelination, cell migration, chemotaxis, MO3.13 oligodendrocytes, receptor trafficking, organotypic slice model

## Abstract

The EBI2 receptor regulates the immune system and is expressed in various immune cells including B and T lymphocytes. It is also expressed in astrocytes in the central nervous system (CNS) where it regulates pro-inflammatory cytokine release, cell migration and protects from chemically induced demyelination. Its signaling and expression are implicated in various diseases including multiple sclerosis, where its expression is increased in infiltrating immune cells in the white matter lesions. Here, for the first time, the EBI2 protein in the CNS cells in the human brain was examined. The function of the receptor in MO3.13 oligodendrocytes, as well as its role in remyelination in organotypic cerebellar slices, were investigated. Human brain sections were co-stained for EBI2 receptor and various markers of CNS-specific cells and the human oligodendrocyte cell line MO3.13 was used to investigate changes in EBI2 expression and cellular migration. Organotypic cerebellar slices prepared from wild-type and cholesterol 25-hydroxylase knock-out mice were used to study remyelination following lysophosphatidylcholine (LPC)-induced demyelination. The data showed that EBI2 receptor is present in OPCs but not in myelinating oligodendrocytes in the human brain and that EBI2 expression is temporarily upregulated in maturing MO3.13 oligodendrocytes. Moreover, we show that migration of MO3.13 cells is directly regulated by EBI2 and that its signaling is necessary for remyelination in cerebellar slices post-LPC-induced demyelination. The work reported here provides new information on the expression and role of EBI2 in oligodendrocytes and myelination and provides new tools for modulation of oligodendrocyte biology and therapeutic approaches for demyelinating diseases.

## 1. Introduction

The Epstein–Barr virus-induced gene 2 (EBI2, GPR183) is highly expressed in the immune tissue and cells where it regulates innate and adaptive immune responses [1,2]. Accordingly, it is highly expressed in B and T lymphocytes and its signaling is implicated in a range of autoimmune diseases including type 1 diabetes, inflammatory bowel disease, rheumatoid arthritis and multiple sclerosis (MS) [3,4,5,6,7,8]. Specifically in MS, EBI2 was abundantly present in the human brain in infiltrating macrophages and various subsets of lymphocytes in the brains of MS patients [7]. EBI2 expression and function in non-immune tissue and cells were also demonstrated in bone osteoclasts, hepatocytes and astrocytes [9,10,11]. Human and mouse astrocytes are the only brain-specific cells in which EBI2 protein and function has so far been shown [11,12].

The receptor expression and function seem particularly important under neuroinflammatory conditions. For instance, treatment with bacterial lipopolysaccharide (LPS) induces sharp downregulation of EBI2 in primary mouse astrocytes and release of various oxysterols including the EBI2 ligand 7α,25-dihydroxycholesterol (7α,25OHC) [13]. In primary human macrophages, LPS treatment leads to temporary upregulation of the receptor and release of oxysterols [14]. Other factors such as the pro-inflammatory cytokines IL1β and IL23 upregulate and sustain EBI2 expression, while TGFβ/IL6 suppress EBI2 expression in differentiating T helper cells [7]. Importantly, EBI2 expression increases during B cell maturation and after interaction with T helper cells [15,16,17]. In the experimental autoimmune encephalomyelitis (EAE) model of MS, EBI2 expression increased in pathogenic Th17 cells and was substantially lower in naïve cells in comparison to memory T cells [7,8]. In untreated relapsing-remitting MS (RRMS), a similar level of EBI2 expression was found in patients and the healthy controls; however, there was significant variability in EBI2 receptor levels found in CD4+ and CD1 T cells in the MS patients’ samples [18]. Moreover, treatment with an anti-α4-integrin humanized antibody (natalizumab) induces a 3-fold increase in EBI2 expression only in CD4+ T cells, an effect not observed after another MS treatment—dimethyl fumarate [8].

The endogenous EBI2 ligand 7α,25OHC is enzymatically synthesized from cholesterol with cholesterol 25-hydroxylase (CH25H) and 25-hydroxycholesterol 7-alpha-hydroxylase (CYP7B1) [19,20]. Upon activation with 7α,25OHC, EBI2 exhibits chemotactic properties and induces chemotaxis of various EBI2 positive cells. Primary B and T lymphocytes from healthy and MS patients, primary human macrophages and mouse and human astrocytes were all shown to migrate specifically in response to EBI2 activation with 7α,25OHC [7,8,11,14,18]. In addition to the studies investigating the effects of exogenously added 7α,25OHC on cellular chemotaxis in vitro, we also demonstrated that macrophages migrate towards astrocyte-released 7α,25OHC [13]. In the EAE model, the concentration of 7α,25OHC in the central nervous system (CNS) was sharply elevated as a result of increased release of CH25H and CYP7B1 enzymes by microglia and CNS infiltrating immune cells, respectively [7]. Wanke and colleagues also showed that EBI2 enhances CNS infiltration of EBI2 expressing pathogenic Th17 cells in the passive EAE. Furthermore, in the EAE model, Chalmin and colleagues found that deficiency of the CH25H enzyme limits trafficking of CD4+ T cells to the inflamed CNS [18]. Similarly, primary human lymphocytes from MS patients exhibit strong migratory response towards 7α,25OHC in vitro. This migratory response was potentiated in lymphocytes from natalizumab treated patients [8].

The roles of EBI2 in the EAE model, MS and astrocytes have been investigated; however, the key question about its expression and function in oligodendrocytes, the cells most affected in MS, remains to be answered. We showed before that EBI2 is involved in myelination under normal and demyelinating conditions. In our studies, normal myelin development was delayed in mice deficient in EBI2 [21]. In organotypic cerebellar slices challenged with lysophosphatidylcholine (LPC), simultaneous treatment with the EBI2 agonist 7α,25OHC prevented demyelination. This protective effect was absent in EBI2-deficient slices and in the EBI2 antagonist NIBR189 treated slices, indicating a direct involvement of EBI2 signaling in protection from demyelination. Importantly, long-term inhibition of EBI2 signals with the antagonist NIBR189 leads to the inhibition of normal myelin development in organotypic cerebellar slices.

Here, to investigate the function of EBI2 in oligodendrocytes and remyelination, we first examined its expression in oligodendrocyte progenitor cells (OPCs, PDGFRα+ cells) and myelinating oligodendrocytes (myelin basic protein (MBP)+ cells) in the human brain. We then inspected the changes in EBI2 expression during oligodendrocyte maturation and its chemotactic properties in MO3.13 oligodendrocytes. Finally, the receptor’s involvement in protection from demyelination in CH25H-deficient mice and remyelination in the wild-type (WT) mice was also explored in the cerebellar organotypic slice model.

## 2. Results

### 2.1. EBI2 Receptor Is Expressed in OPCs in the Human Brain

**Figure 1:**
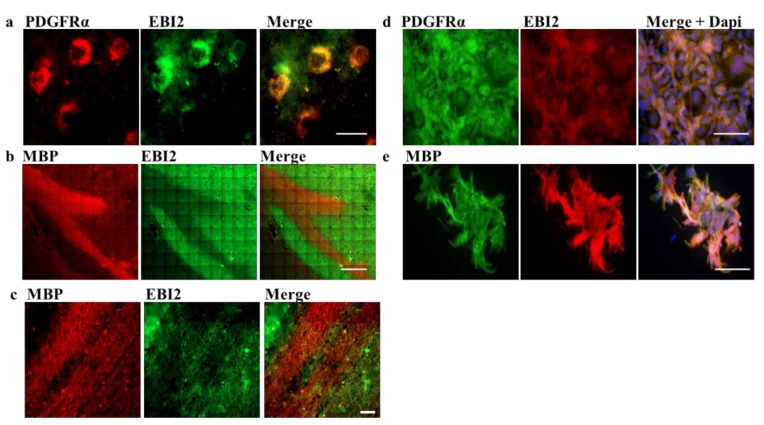
EBI2 is expressed in OPCs in the human brain. (**a**) EBI2 is expressed in the human brain in OPCs as indicated by double staining with anti-PDGFRα (red) and anti-EBI2 (green) antibody. Scale 20 μm. (**b**) EBI2 expression is not detectable in mature myelin sheets. The images show MBP positive (red) myelinated tracts with no EBI2 (green) staining. MBP images were taken at low magnification to show a large portion of the myelinated nerve tract (corpus callosum). Scale 600 μm. (**c**) Higher magnification of the corpus callosum myelinated tracts. Scale 40 μm. Representative images of N = 3 human brains. (**d**) EBI2 is expressed at protein level in PDGFRα-expressing and (**e**) MBP-expressing human MO3.13 oligodendrocytes (N = 3). Scale 100 μm.

EBI2 receptor’s involvement in diseases of the CNS, including MS, was demonstrated before [7,8,22]. However, EBI2 expression in brain cells has only so far been shown in primary human and mouse astrocytes [11]. Here, to investigate EBI2 expression in the CNS-specific cells, we co-stained human brain sections for EBI2 receptor and the markers of OPCs (PDGFRα), mature oligodendrocytes (MBP), astrocytes (GFAP), microglia (Iba1) and neurons (NFH). The data showed, for the first time, that EBI2 is present in OPCs (PDGFRα-positive cells) in the human brain (Figure 1a). EBI2 was not, however, present in mature myelin as indicated by no EBI2 staining in the MBP-positive myelin sheets (MBP protein in myelinating oligodendrocytes is predominantly localized to cell extensions [23]) (Figure 1b,c). In human MO3.13 oligodendrocytes, the receptor was present in PDGFRα- and MBP-expressing oligodendrocytes (Figure 1d,e). The data also confirmed previously reported EBI2 expression in astrocytes [11] and, for the first time, in microglia (Iba1+) in the human brain (Supplementary Figure S1a,b). No EBI2 in neurons (NFH+) was found in the human brain sections where distinct (non-overlapping) NFH and EBI2 staining was observed (Supplementary Figure S1c). High EBI2 expression (Ct values of 23) was also confirmed at the mRNA level in primary human OPCs (hOPCs) and exceptionally high (27-fold higher than in hOPCs, Ct values of 19) expression was found in primary human microglial cells (hMicros) (Supplementary Figure S1d). Protein dot blots confirmed EBI2 in primary hOPCs and MO3.13 oligodendrocyte lysates and trace amounts of EBI2 receptor in primary human neuron cell lysate (Supplementary Figure S1e).

### 2.2. MO3.13 Oligodendrocytes Temporarily Upregulate EBI2 Receptor during Maturation

**Figure 2:**
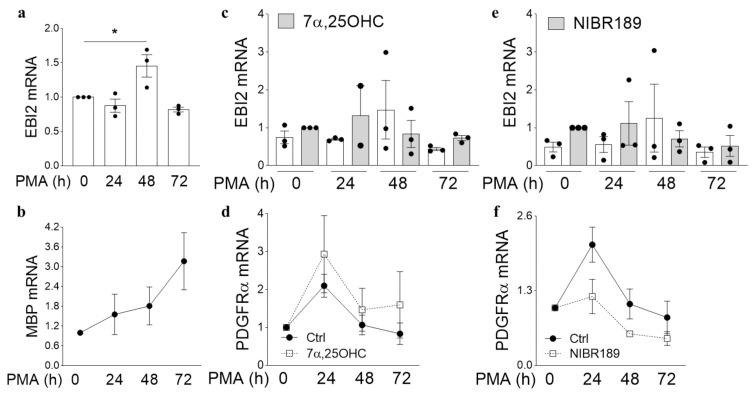
MO3.13 oligodendrocytes temporarily upregulate EBI2 during maturation. (**a**) EBI2 expression temporarily increases after initiation of MO3.13 oligodendrocyte maturation with PMA (150% +/− 16% PMA 0 h vs. PMA 48 h, *p* = 0.0258) and returns to baseline thereafter (82% +/− 3.4% PMA 0 h vs. PMA 72 h, *p* = 0.4553). One-way analysis of variance with Dunnett’s post-hoc tests, *p* = 0.0063, N = 3, * *p* < 0.05. (**b**) Treatment of MO3.13 oligodendrocytes with PMA induces their maturation as indicated by increasing transcripts of MBP mRNA over the period of 72 h. N = 10. (**c**) Simultaneous treatment of MO3.13 oligodendrocytes with PMA and the EBI2 agonist 7α,25OHC inhibits the maturation-induced EBI2 upregulation) and (**d**) is not essential for oligodendrocyte maturation as can be seen by similarly decreasing PDGFRα transcripts in PMA (Ctrl) and 7α,25OHC treated cells. Two-way analysis of variance, *p* > 0.05, N = 3. (**e**) Similarly, Treatment of MO3.13 oligodendrocytes with PMA and the EBI2 antagonist NIBR189 does not induce EBI2 expression. One-way analysis of variance (*p* = 0.6074, N = 3). (**f**) Antagonism of EBI2 signaling does not inhibit maturation of PMA-stimulated MO3.13 oligodendrocytes. Two-way analysis of variance, *p* > 0.05, N = 3.

EBI2 expression in the cells of the immune system increases during B cell maturation and after interaction with T helper cells [15,16,17]. Here, to explore changes in EBI2 expression in the cells of the CNS, we investigated EBI2 expression in MO3.13 oligodendrocytes during maturation induced with PMA. The data showed that, similarly to B cells, EBI2 expression temporarily increases in MO3.13 oligodendrocytes as they begin to mature (Figure 2a). PMA-induced maturation of MO3.13 oligodendrocytes is indicated by steadily increasing MBP expression (Figure 2b). We then examined whether simultaneous activation of EBI2 with exogenously added 7α,25OHC or inhibition of its signaling with the antagonist NIBR189 during PMA-induced maturation affects oligodendrocyte maturation or receptor expression. The results indicated that co-treatment of MO3.13 oligodendrocytes with PMA and the agonist 7α,25OHC attenuate the maturation-induced increase in EBI2 expression (Figure 2c) and have no effect on oligodendrocyte maturation, as indicated by similarly decreasing PDGFRα transcripts in the control and 7α,25OHC-treated cells (Figure 2d). Similar data were obtained for the cells co-treated with PMA and the EBI2 antagonist NIBR189 (Figure 2e,f). These data indicate that EBI2-mediated signaling may be involved in MO3.13 oligodendrocyte maturation but is non-essential for maturation to proceed normally. The degree and nature of EBI2 involvement in oligodendrocyte maturation needs to be further investigated. Importantly, this is the first report of EBI2 upregulation in a non-inflammatory/non-immune setting suggesting that EBI2 may play other important roles in the CNS.

### 2.3. EBI2 Signaling Regulates MO3.13 Oligodendrocyte Migration

**Figure 3:**
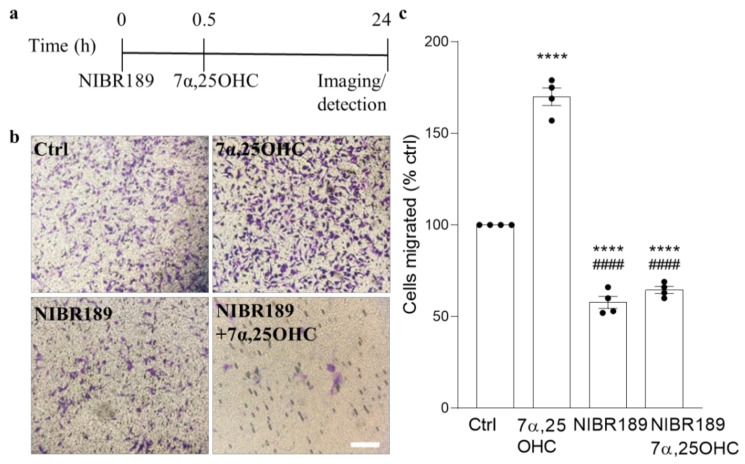
EBI2 signaling regulates migration of human MO3.13 oligodendrocytes. (**a**) Graphical illustration of the experimental setup in the migration assay and (**b**) representative images showing the number of cells (purple) that migrated across the Boyden chamber membrane. (**c**) Quantification of the extent of cellular migration shows a significant increase in the number of migrating MO3.13 oligodendrocytes towards the EBI2 agonist, oxysterol 7α,25OHC (170% +/− 4.8% vs. ctrl, *p* < 0.0001). Pre-treatment (30 min) with EBI2 antagonist NIBR189 inhibits 7α,25OHC-induced OPC migration (38.2% +/− 1.1% vs. 7α,25OHC, *p* < 0.0001 and 65% +/− 1.9% vs. ctrl, *p* < 0.0001) clearly indicating EBI2-driven induction of MO3.13 oligodendrocyte migration. One-way analysis of variance with Sidak’s post-hoc tests, *p* < 0.0001, N = 4 independent experiments, **** *p* < 0.0001 vs. Ctrl, #### *p* < 0.0001 vs. 7α,25OHC. Scale 50 μm.

EBI2 is a chemotaxis-inducing receptor in macrophages, lymphocytes (B and T), dendritic cells and astrocytes [8,11,14,19]. In MS, EBI2 was shown to modulate the migration of CD4+ T cells in patients receiving natalizumab therapy [8] and in the EAE model, the influx of autoreactive lymphocytes into the CNS [7,18]. Here, after we demonstrated EBI2 expression in OPCs in the human brain and MO3.13 oligodendrocytes, we investigated whether the expressed receptor is functional and induces oligodendrocyte migration (Figure 3a). The data indicated a significant 1.7-fold induction of oligodendrocyte migration towards the EBI2 agonist, the oxysterol 7α,25OHC, as shown by the large number of cells that crossed the Boyden chamber membrane (Figure 3b). Migration of these cells was not induced by the antagonist treatment (NIBR189) alone but the 7α,25OHC-induced chemotaxis was inhibited by the antagonist, demonstrating a direct effect of EBI2 signaling on the migratory behavior of MO3.13 oligodendrocytes (Figure 3c).

### 2.4. Antagonism of EBI2 Signaling Inhibits Remyelination in Cerebellar Slices

**Figure 4:**
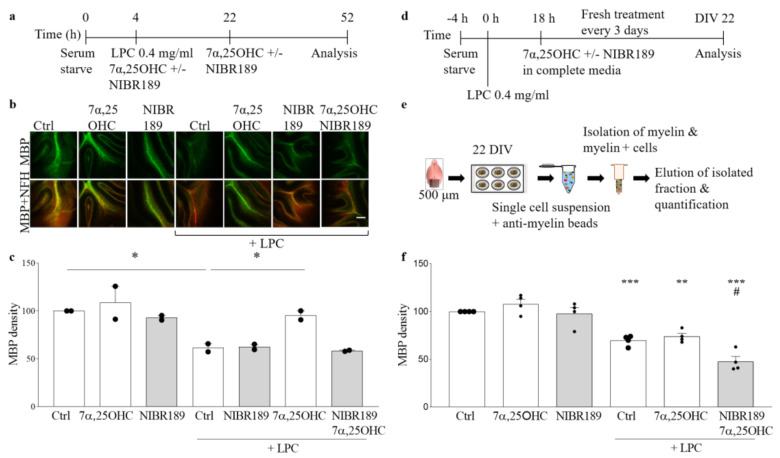
Antagonism of EBI2 signaling inhibits remyelination in cerebellar slices. (**a**) The experimental design is shown on a schematic diagram. (**b**) Exemplary images from confocal microscope show organotypic cerebellar slices made from CH25H KO mice immunostained with MBP (green) and NFH (red). Scale 100 μm (**c**) A complementary bar graph shows quantification of MBP density. Addition of the EBI2 agonist 7α,25OHC at the same time as LPC protects from demyelination (158.2% +/− 8.2% vs. LPC). However, simultaneous addition of the agonist and the antagonist NIBR189 blocks the agonist-mediated protective effects (100.8% +/− 0.9% vs. LPC) indicating direct oxysterol/EBI2 signaling involvement in protection from chemically induced demyelination. Data presented as mean +/− SEM (N = 2 independent experiments, 6 mice per experiment), one-way analysis of variance with Sidak’s post-hoc test, * *p* < 0.05, vs. corresponding control. (**d**) Graphical representation of experimental setup and (**e**) the sequence of tissue preparation and analysis with the MBP quantification method. (**f**) Treatment with LPC for 18 h induces significant loss of myelin proteins indicative of demyelination in organotypic cerebellar slices (70% +/− 2.7% ctrl vs. LPC, *p* < 0.001). Chemically induced demyelination is still observed after 22 days of recovery in complete media. Treatment with EBI2 agonist 7α,25OHC alone does not enhance remyelination above the spontaneous remyelination levels observed in the control slices (74% +/− 3.2% ctrl vs. LPC/7α,25OHC, *p* = 0.003). However, simultaneous inhibition of EBI2 signaling with the antagonist NIBR189 significantly inhibited remyelination (68.6% +/− 7.5% LPC vs. LPC/7α,25OHC/NIBR189, *p* = 0.001 and vs. ctrl, *p* < 0.01) indicating EBI2-mediated regulation of remyelination. One-way analysis of variance with Sidak’s post-hoc tests, N = 4 independent experiments, # *p* < 0.001 vs. LPC, ** *p* < 0.01 vs. Ctrl and *** *p* < 0.001 vs. Ctrl.

We showed before that simultaneous co-treatment of slices with LPC and 7α,25OHC protects from LPC-induced demyelination in cerebellar slices [21]. In these experiments, protection from demyelination was absent in slices prepared from EBI2-deficient mice and when EBI2 receptors were blocked with the antagonist demonstrating a direct EBI2 involvement in these processes. The rate-limiting enzyme CH25H is required for the synthesis of the EBI2 agonist 7α,25OHC. Importantly, CH25H genes were upregulated in microglia in the mouse cuprizone model during de- and remyelination presumably to facilitate remyelination [24]. To investigate the involvement of oxysterol signaling in protection from demyelination, organotypic slices of cerebellum were made from CH25H KO mice and treated with LPC and 7α,25OHC and/or NIBR189 (Figure 4a) and then immunostained with anti-MBP and anti-NFH antibodies (Figure 4b). The data showed that myelination in cerebellar slices prepared from P10 CH25H KO pups proceeds normally, indicating that enzyme CH25H is non-essential for normal myelination (Figure 4b,c). However, under demyelinating conditions, exogenous addition of 7α,25OHC attenuated LPC-induced demyelination in CH25H KO cerebellar slices and co-treatment with NIBR189 blocked the 7α,25OHC-mediated effects (Figure 4c). These data further confirm that oxysterol/EBI2-mediated signaling protects from demyelination under demyelinating conditions as we have shown before in the WT and EBI2 KO mice [21]. Next, we asked the question whether EBI2 signaling plays a role in spontaneous remyelination after damage, that is, where the EBI2 agonist and antagonist are added post-LPC to demyelinated slices. To accomplish this task, we developed a novel, more objective method of myelin content quantification using anti-myelin magnetic beads (Figure 4d–f). The new method was validated against the conventional method of immunohistochemical staining used here for myelination analysis in CH25H slices (Figure 4a–c and Supplementary Figure S2. The data obtained with the new method of myelin quantification showed that post-LPC treatment with the EBI2 agonist 7α,25OHC alone did not have any additional effects on spontaneous remyelination after the removal of LPC (Figure 4f). However, long-term antagonism of EBI2 signaling post-LPC treatment significantly inhibited spontaneous remyelination in this model. Altogether, these results indicate that EBI2 signaling is indeed directly involved in myelination in the presence of a demyelinating agent such as LPC in an ex vivo model. If these results are replicated in vivo, modulation of the oxysterol/EBI2 pathway may become a novel drug target for myelinating therapies.

### 2.5. 7α,25OHC-Induced EBI2 Internalization Is βArrestin2 Dependent

**Figure 5:**
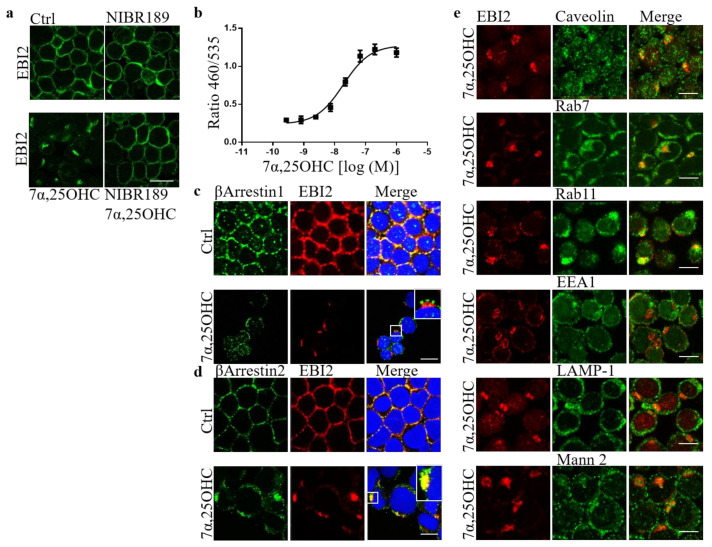
7α,25OHC induces EBI2 internalization. (**a**) 7α,25OHC induces EBI2 (green) internalization in U937 human monocytes. Treatment with the EBI2 antagonist (NIBR189) blocks 7α,25OHC-induced internalization. N = 3, scale 20 μm. (**b**) Tango assay in EDG8-bla U2OS cells containing the human EBI2 (GPR183) shows recruitment of βArrestins after hEBI2 activation by 7α,25OHC. Data presented as mean +/− SEM, N = 3. (**c**) βArrestin1 (green) does not interact with EBI2 receptor (red) as observed by separate red and green staining. Scale 20 μm (**d**) βArrestin2 (green) colocalizes with EBI2 (red) indicating an interaction between the two proteins (yellow color). Representative confocal images, N = 3, Scale 20 μm. (**e**) Internalized EBI2 (red) colocalizes with caveolin, Rab7 and Rab11 (yellow color) and does not colocalize with EEA1, LAMP-1 and Mannosidase-2 proteins. N = 3, scale 10 μm.

We showed before that long-term inhibition of EBI2 signaling in cerebellar slices prepared from P10 mice attenuates normal myelin development [21]. We also showed that 7α,25OHC induces EBI2 receptor internalization in U937 monocytes [21]. To shed some light on the EBI2-mediated spontaneous remyelination post-LPC reported here in Figure 4d–f we examined the dynamics of EBI2 expression and trafficking. We hypothesized that long-term antagonism of EBI2 leads to receptor internalization and degradation leading to the loss of 7α,25OHC-induced signaling needed for remyelination. In our previous reports, we showed that 7α,25OHC induced internalization in U937 monocytes and EBI2 antagonist NIBR189 treatment did not affect receptor membrane density [21]. Here, we extended the analysis of receptor trafficking in these cells by examining various intracellular organelle markers. The data confirmed that EBI2 is internalized after activation with the agonist, 7α,25OHC, and that the receptor’s expression remains unchanged after incubation with the antagonist NIBR189 alone. However, here we additionally show that when the cells are treated simultaneously with the agonist and antagonist, the receptor internalization is inhibited (Figure 5a). We then investigated the trafficking of the internalized receptor and found that EBI2 internalization is βArrestin2-dependent (Figure 5b–d). Images showed no colocalization with caveolin, suggesting receptor-dependent endocytosis. After internalization, the receptor travels to late endosomes and the Golgi apparatus and is most likely recycled back to the cell membrane, as shown by the interaction of internalized EBI2 with late endosomal and recycling proteins (Rab7, Rab11) and no interaction with lysosomal proteins (LAMP-1, Mannosidase-2) nor with EEA1 protein, a marker of early endosomes (Figure 5e). These data show that short-term activation of EBI2 with the agonist 7α,25OHC induces a clear internalization of the receptor most likely followed by the return of the receptor to the cell membrane. Whether the receptor is recycled back to the surface or degraded after long-term stimulation remains to be elucidated. However, our investigations suggest that after short-term activation with 7α,25OHC, the receptor density at the cell membrane does not return to the control levels even after 7 h of a wash-out period (data not shown).

## 3. Discussion

The EBI2 receptor’s role in MS pathophysiology and myelination were studied in human and animal models before [7,8,18,21]. However, its expression and function in oligodendrocytes have never been investigated. Here, we studied EBI2 expression and function in oligodendrocytes in the human brain and MO3.13 cells and its involvement in myelination in organotypic slices of the cerebellum. The data, for the first time, showed EBI2 in OPCs and microglia and confirmed the receptor’s presence in astrocytes in the human brain. Our studies showed that oligodendrocytes upregulate EBI2 expression during maturation but that EBI2-signaling is non-essential for oligodendrocyte maturation to proceed normally.

Importantly, to our knowledge, this is the first report of EBI2 upregulation in a non-inflammatory or non-immune setting suggesting that EBI2 may have other roles to play in the CNS beside immune regulation. The data reported here also showed that EBI2 in MO3.13 oligodendrocytes is functional and that activation of EBI2 induces migration of MO3.13 oligodendrocytes towards higher concentrations of 7α,25OHC. The ability to induce migration in immature oligodendrocytes may prove very useful in treating demyelinating diseases where OPCs need to migrate towards the demyelinated area. Perhaps the temporary upregulation of EBI2 in maturing oligodendrocytes is linked to cellular migration. By upregulating EBI2, the cells may migrate to and better position themselves in demyelinated areas where oxysterol concertation is increased as a result of cholesterol release from damaged myelin sheets and synthesis by astrocytes and other infiltrating immune cells.

In the organotypic cerebellar slice model, induction of EBI2 signaling with its agonist 7α,25OHC did not have any additional effect on normally occurring remyelination post-LPC-induced demyelination. However, when EBI2 signaling was blocked with the antagonist, remyelination was inhibited. These data show that even though increased activation of EBI2 does not enhance normally occurring remyelination it is essential to proceed normally. Oxysterols, including EBI2 agonist 7α,25OHC, are present in the serum in slice media and are released by the injured tissue post-LPC-treatment. These endogenous oxysterols induced EBI2 signaling in the control slices and thus no additional effect was observed after further addition of exogenous 7α,25OHC. Remyelination was not enhanced above the control levels in 7α,25OHC-treated slices most likely as a result of the limits in the inherent capacity to synthesize myelin. However, long-term antagonism of EBI2 revealed that its signaling is essential for remyelination in this model as the antagonist blocked EBI2 signaling induced by the endogenous as well as the exogenously added oxysterols and inhibited remyelination. Furthermore, persistent antagonism of EBI2 most likely inhibited EBI2-mediated OPC migration towards the demyelinated tissue, where oxysterol levels are increased.

Our previous work showed that in EBI2-deficient mice, normal myelination is delayed in the post-natal period [21]. We also discovered before that long-term and persistent antagonism of EBI2 inhibits normal myelin development in cerebellar slices from P10 mice [21]. We confirmed here that EBI2 is indeed internalized after 7α,25OHC treatment and showed that its internalization is βArrestin2 dependent. The trafficking and intracellular organelle data suggested that after short-term stimulation, EBI2 is recycled back to the cell membrane. We could not show with the current model that long-term EBI2 antagonism or stimulation leads to receptor downregulation and degradation and needs to be clarified. Altogether, the results reported here demonstrate a direct role of EBI2 signaling in remyelination. The EBI2 agonist 7α,25OHC is an oxysterol and oxysterols are derivatives of cholesterol, which is the main component of myelin. EBI2 signaling might therefore be necessary for correct cholesterol/myelin synthesis under normal and demyelinating conditions.

**Figure 6:**
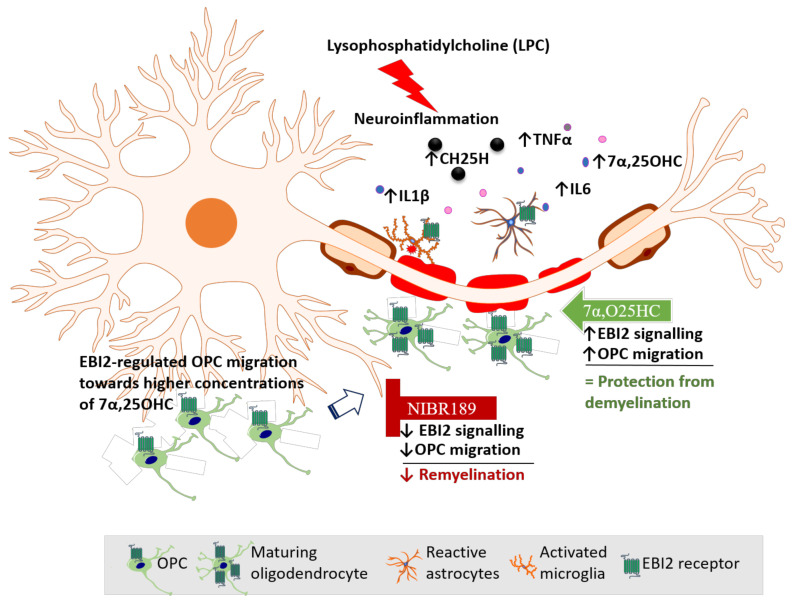
Mechanism of action of EBI2-mediated regulation of OPC migration and remyelination. LPC-treatment induces demyelination and inflammation in cerebellar slices. There is a marked rise in the pro-inflammatory cytokine (IL6, TNFα, IL1β) levels and oxysterols (e.g., 7α,25OHC), released by activated EBI2+ astrocytes and microglia that respond to myelin damage. OPCs upregulate EBI2 and begin to migrate towards affected areas in EBI2-dependent manner (in response to increased concentration of 7α,25OHC) and EBI2-independent manner (in response to other chemotactic molecules released during inflammation). Once at the site of injury, OPCs begin to mature and remyelinate the damaged tissue. Exogenous addition of 7α,25OHC (green arrow) increases EBI2 signaling, enhances OPC migration and supports remyelination with (no additional effect). Addition of NIBR189 (red sign) blocks EBI2 signaling, reduces OPC migration and leads to inhibition of remyelination.

Based on our data reported here and elsewhere, we propose a model of EBI2 function in the CNS (Figure 6). In this model, LPC challenge induces inflammation and demyelination in cerebellar slices. Activated EBI2+ astrocytes and microglia respond to myelin damage and release pro-inflammatory cytokines and oxysterols. EBI2-expressing OPCs upregulate EBI2 and begin to migrate towards the affected areas in EBI2-dependent manner in response to increased concentration of 7α,25OHC and EBI2-independent manner in response to other chemotactic molecules. Once at the site of injury, OPCs mature, start to synthesize myelin and downregulate EBI2. Exogenous addition of 7α,25OHC increases migration of OPCs while inhibition of EBI2 signaling with NIBR189 leads to downregulation of EBI2 signaling and inhibition of OPC migration, which negatively affects remyelination.

## 4. Materials and Methods

### 4.1. Mice

The Ch25h(−/−) knock-out (KO) mice (C57BL/6) were provided by Dr. David W. Russell (University of Texas Southwestern, TX, USA) [25]. All animals were kept in filter-top cages under specific pathogen-free conditions with ad libitum access to standard diet and water. All animal tissue was harvested according to the national and institutional ethical guidelines.

### 4.2. Immunohistochemistry of Human Brain Sections

Post-mortem human brain sections from MS donors preserved in formalin were received from the Rocky Mountain MS Center Tissue Bank (Englewood, CO, USA) after approval by the Medical University of Gdansk (Poland) bioethics committee (NKBBN/253/2018). Portions of 1 cm × 1 cm containing white and grey matter from periventricular region were removed and soaked in 15% followed by 30% sucrose solution to dehydrate. The Tissue-Tek OCT Compound (4583, Sakura, The Netherlands)-embedded brain portions were cut into 30-micron thick slices on a cryostat. The non-specific binding was reduced by incubation in 10% normal goat serum (NGS) in 0.3% triton-x in PBS (blocking solution) for two hours at RT. Then, the sections were washed 3 × 15 min in PBS and incubated in a primary antibody cocktail o/n in the blocking solution. After further washes, the tissue was incubated o/n in secondary antibody cocktail (Cy3 goat anti-rabbit (Abcam, ab6939, RRID:AB_955021) or/and Alexa 488 goat anti-mouse (ThermoFisher, A-11001, RRID:AB_2534069)) after which it was washed, inserted onto glass cover slides and imaged using an automated Z1 system (Zeiss, Oberkochen, Germany) using the Zeiss Zen 3.1 software (Zeiss, Oberkochen, Germany). Primary antibodies used were: Rabbit PDGFRα (ThermoFisher, PA5-16571, RRID: AB_10981626), rabbit MBP (CellSignaling, 78896, RRID: AB_2799920), chicken NFH (Millipore, AB5539, RRID:AB_11212161), rabbit GFAP (CellSignaling, 12389, RRID:AB_2631098, mouse EBI2 (clone 57C9B51C9, Novartis, Basel, Switzerland [7]).

### 4.3. Organotypic Slice Culture, Treatments and Immunohostochemistry

Organotypic cerebellar slices were made from 10-day-old (P10) CH25H−/− KO or wild-type (WT) C57BL/6 mice according to previously published protocol [21]. Briefly, pups were decapitated with scissors, the skull was opened, and the cerebellum removed and placed in cold Opti-MEM medium (11058021, ThermoFisher, Warsaw, Poland). Parasagittal 400 μm thick slices of the cerebellum were cut using the McIlwain tissue chopper WPI, Friedberg, Germany). Several (4–6) slices were cultured on a single insert (Merck, Millicell, PICMORG50) in a humidified incubator at a reduced temperature of 35.5 °C and standard 5% CO_2_ concentration. The growth medium consisted of 50% Opti-MEM, 25% Hanks’ buffered salt solution (HBSS), 25% heat-inactivated horse serum supplemented with Glutamax (2 mM), D-glucose (28 mM), 1% pen/strep, HEPES (10 mM) for 12 days in vitro. After 12 days in vitro, slices were placed in serum free media for 4 h and then again in fresh serum-free media supplemented with LPC (0.4 mg/mL) (Sigma, L-4129) with or without 7α,25OHC (1 μM) and/or NIBR189 (NIBR189, 1 μM) for 18 h. Following the 18 h LPC, 7α,25OHC and/or NIBR189 treatment, the slices were cultured in medium containing 7α,25OHC (1 μM) and/or NIBR189 (1 μM) for further 30 h (a total of 48 h) (Figure 4a,b). When the treatments were finished, the slices were washed twice with PBS and fixed in 4% paraformaldehyde (PFA) for 5 min at RT and permeabilized with 20% ice cold methanol for 5 min. Subsequently, the slices were washed 2 × 5 min in PBS and incubated overnight in the blocking buffer that consisted of Triton-X100 (0.5%), bovine serum albumin (10%, BSA; Sigma Aldrich), NGS (1%). The slices were then incubated overnight at 4 °C with the primary antibodies (rabbit anti-myelin basic protein (MBP, Abcam ab40390, RRID:AB_1141521) and chicken anti-neurofilament heavy chain (NFH), Millipore, AB5539, RRID:AB_11212161) in PBS supplemented with BSA (0.5%) and Tween-20 (0.05%), washed twice in the above solution and incubated with secondary antibodies overnight at 4 °C. Finally, the slices were washed twice with wash solution as was described earlier and mounted on glass cover slides with Tissue-Tek OCT Compound. Confocal images were taken from the central part of the slices and relative fluorescence intensity was analyzed with ImageJ software (Bethesda, MD, USA).

### 4.4. Organotypic Slice Culture, Treatments and Myelin Content Analysis with Myelin-Coated Beads

Cerebella from the WT mice were placed in a stainless-steel brain matrix (AgnTho’s, Lidingö, Sweden) for cutting of 500 μm thick mouse sagittal slices with a sterile blade. The cut slices were moved into a petri dish containing Opti-MEM medium and slices were separated from each other using needles under a microscope. Four slices per condition were cultured on one organotypic insert at 35 °C, 5% CO_2_. Media change was done on day in vitro (DIV) 1, 4 and 7 with media consisting of 50% Opti-MEM, 25% HBSS, 25% horse serum, 1% glucose, 1% HEPES buffer, 1% GlutaMax and 1% pen/strep. At DIV 10, slices were serum-starved with media containing 75% HBSS, 25% Opti-MEM, 1% GlutaMax, 1% glucose, 1% pen/strep and 1% HEPES for 4h before adding LPC 0.4 mg/mL for 18h. After 18h incubation with LPC, media was removed and slices were transferred to serum-containing media with 0.25 µM 7α,25OHC or 100 µM NIBR189 for another 12 days, changing to fresh media with compounds every 3 days (Figure 4d,e). At DIV 22, slices were collected, and brain cells were dissociated using the MACS Neural Tissue Dissociation Kit P (Miltenyi, Bergisch Gladbach, Germany, 130-092-628) according to the accompanying product protocol with the MACS separator. The dissociated cells were incubated with myelin beads (Miltenyi, Bergisch Gladbach, Germany, 130-104-257) and separated using LS columns (Miltenyi, Bergisch Gladbach, Germany, 130-042-401). The amount of myelin was determined using a BCA assay (Thermofisher, Waltham, MA, USA, 23225) as a ratio of myelin/total.

### 4.5. Cell Culture and Differentiation

MO3.13 human oligodendrocyte cell line (RRID:CVCL_D357) was purchased from Tebu-Bio (2018, CLU301, batch 131117 P25) and grown in high glucose DMEM, 10% FBS and 1% pen/strep. Cells were differentiated with 100 nM PMA (Sigma-Aldrich, P1585) with or without the EBI2 agonist oxysterol 7a,25-dihydroxycholesterol (7α,25-OHC) (1 μM) or EBI2 antagonist NIBR189 (1 μM) in T25 flasks for 0–72 h.

U937 cells were cultured in RPMI 1640 medium containing Glutamax, 10% FBS, 1% MEM non-essential amino acids (NEA, Invitrogen, Carlsbad, CA, USA, 11140), 1% sodium pyruvate (Invitrogen, 11360-070), 1% pen/strep and 0.1% 2-Mercaptoethanol (Sigma-Aldrich, St. Louis, MO, USA, M7154) in T75 culture flasks.

### 4.6. Migration Assay

MO3.13 undifferentiated oligodendrocytes were starved for 3 h before treatment in serum-free media. Serum-free DMEM with or without 0.1 µM 7α,25OHC or 1 µM NIBR189 was pipetted in a 24 well-plate. Then, 150 µl cell suspension of 2 million cells per ml were plated in the top chamber of the transwell assay insert (ThermoFisher, 141006, 8 µm pores). After 30 min of incubation with the antagonist, the insert with cells was moved into wells containing the oxysterol with or without NIBR189 for 6 h. After 6 h, the media inside the transwell was discarded, and the cells that did not migrate were removed with a cotton swab. The transwells were put in 400 µL of crystal violet staining solution (Cell Biolabs, San Diego, CA, USA, CBA-100) for 10 min at RT. Then, they were dipped carefully in a beaker with water and left to air dry. When dried, images were taken with a light microscope and transwells were incubated in 200 µL of extraction solution (Methanol, Cell Biolabs, CBA-100) for 10 min on a shaker. Then, the solution was transferred to a 96-wellplate in order to read absorbance at 590 nm.

### 4.7. Immunocytochemistry

MO3.13 oligodendrocytes in 8-well imaging plates (Milicell, Millipore) were washed with cold PBS, fixed for 10 min on ice in 4% PFA and then permeabilized for 60 s in coldmethanol (100%). The non-specific binding was blocked in the incubation in 0.5% NGS, 1% BSA, 0.1% tween-20 in PBS for 60 min and then incubated o/n at 4 °C with primary antibodies in PBS containing BSA (0.5%), tween-20 (0.05%). The following primary antibodies were used: Rabbit PDGFRα (ThermoFisher, PA5-16571, RRID: AB_10981626), rabbit MBP (CellSignaling, 78896, RRID: AB_2799920), mouse EBI2 (clone 607B, Novartis). The cells were then washed with PBS 3 × 5 min and incubated for 60 min with the following secondary antibodies: Anti-rabbit Alexa 488 and anti-mouse 546. Then they were washed again, incubated with Hoechst (ThermoFisher, H1399) for 10 min, washed and imaged with Nikon Eclipse Ti fluorescent microscope (Tokyo, Japan).

The U927 monocytes were washed with PBS and treated for 1 h at 37 °C with 7α,25OHC (10 μM) or NIBR189 (10 μM). After the treatment, the cells were spun in a Cytospin 4 Cytocentrifuge (Thermo Scientific, A78300003) and adhered to glass slides. Subsequently, the U937s on glass slides were washed with PBS and fixed with fixation/permeabilisation kit (BD Cytofix/Cytoperm, 554714) according to the manufacturer’s protocol (20 min on ice). The monocytes were then incubated for 1 h in a blocking solution consisting of PBS, 0.1% tween-20 and 1% BSA. The cells were then incubated overnight at 4 °C with the primary antibodies in 0.05% tween-20 and 0.5% BSA in PBS, washed twice and incubated with secondary antibodies for 1 h in the same solution. The primary antibodies used were mouse EBI2 (clone 57C, Novartis), rabbit Rab11 (Invitrogen, 71-5300, RRID:AB_87868), rabbit EEA1 (Abcam, ab2900, RRID:AB_2262056), rabbit caveolin (BD, 610060, RRID:AB_397472), rabbit LAMP-1 (Abcam, ab24170, RRID:AB_775978), rabbit Rab7 (CellSignaling, D95F2, RRID:AB_1904103), rabbit mannosidase2 (Abcam, ab136497, RRID:AB_722896), rabbit βArrestin 1 (Abcam, ab32099, RRID:AB_722896) and goat βArrestin 2 (Abcam, ab31294, RRID:AB_2060265). The secondary antibodies used were anti-rabbit Alexa 488 (Invitrogen, A11008, RRID:AB_143165), anti-goat Alexa 488 (Invitrogen), anti-mouse Alexa 633 (Invitrogen, A21052, RRID:AB_2535719). After two subsequent washes, the U973s were incubated with Hoechst for 10 min, washed twice again, covered in PBS and kept covered in aluminum foil at 4 °C until imaged. The Zeiss LSM 700 confocal microscope was used to take images of the cells.

### 4.8. Real-Time Quantitative Polymerase Chain Reaction (RT-qPCR)

Human OPC cDNA extracted from normal primary cells was purchased from ScienCell (1604, Carslbad, CA, USA). Human microglia cell total RNA extracted from primary cells was purchased from Celprogen (37089RNA, Torrance, CA, USA). Treated MO3.13 oligodendrocytes were washed in PBS, scraped, spun for 10 min at maximum speed and resuspended in 150 μL of lysis buffer (Sigma-Aldrich, RTN70-1KT). Total RNA was extracted using the GenElute total RNA kit (Sigma-Aldrich, RTN70-1KT). Isolated total RNA was frozen at −20 °C until used. Total RNA was reverse-transcribed with cDNA transcription kit (ThermoFisher, 4368814) according to the manufacturer’s instructions. RT-qPCR was performed with TaqMan master mix (ThermoFisher, 4444556) using the LightCycler 480 (Roche, Basel, Switzerland) according to the standard protocol. FAM dye-labelled TaqMan (Applied Biosystems, Foster City, CA, USA) probes were used in all experiments. The relative mRNA expression using the ΔΔCt method was calculated from absolute quantification after normalization to the reference gene.

### 4.9. βArrestin Assay

The Tango assay (Invitrogen, Carlsbad, CA, USA) is a cell-based system for detection of GPCR activation and βArrestin interaction. The assay was run on EBI2-bla U2OS cells (Invitrogen, K1828A), which express the human EBI2 receptor associated with a TEV protease site and a Gal4-VP16 transcription factor stably integrated into the Tango GPCR-bla U2OS parental cell line. The parental cells stably express a βArrestin/TEV protease fusion protein and the β-lactamase reporter gene under the control of a UAS response element. The EBI2-bla U2OS cells were grown in McCoy’s 5A media (Invitrogen, 36600-088) supplemented with 10% dialyzed FBS (Invitrogen, 26400-036), 0.1 mM NEAA, 25 mM Hepes, 200 µg/mL Zeocin (Invitrogen, R250-01), pen/strep. The experiments were conducted according to manufacturer’s protocol, in brief: EBI2-blaU2OS cells in Freestyle Expression media (Invitrogen, 12338-018) were placed in 384 clear bottom/black walls plates and incubated for 48 h at 37 °C. On the day of the experiment, the cells were treated with 7α,25OHC for 16 h in the incubator. Subsequently, a substrate mixture was loaded in the absence of direct strong light and incubated for another 2 h at RT in the dark. The fluorescence was read with Envision (PerkinElmer, Waltham, MA, USA) plate reader.

### 4.10. Statistical Analysis

Statistical analysis was performed on GraphPad Prism 8 using one-way analysis of variance (ANOVA) followed by Sidak’s multiple comparisons post hoc tests for comparisons of differences between pre-selected groups or Dunnett’s post hoc test for comparisons of the control with the mean of every other column. Data are shown as means ± standard error of the mean (SEM). Where appropriate, *p* values are written in the figure legends, and group comparisons derived from post hoc analysis are given in the figures with significant effects indicated by asterisks: * *p* ≤ 0.05, ** *p* ≤ 0.01, *** *p* ≤ 0.001, **** *p* ≤ 0.0001.

## 5. Conclusions

This work reveals the essential role of EBI2 receptor in oligodendrocyte biology and remyelination. It also proposes a novel mechanism where EBI2-signaling first induces chemotaxis of OPCs towards demyelinated tissue and then, in maturing OPCs, supports myelination.

## Data Availability

The datasets generated during and/or analysed during the current study are available from the corresponding author on reasonable request.

## Supplementary Materials

The following are available online at https://www.mdpi.com/article/10.3390/ijms22094342/s1.

## References

[1] Baptista A.P., Gola A., Huang Y., Milanez-Almeida P., Torabi-Parizi P., Urban J.F., Shapiro V.S., Gerner M.Y., Germain R.N. (2019). The Chemoattractant Receptor Ebi2 Drives Intranodal Naive CD4+ T Cell Peripheralization to Promote Effective Adaptive Immunity. Immunity.

[2] Lu E., Cyster J.G. (2019). G-protein coupled receptors and ligands that organize humoral immune responses. Immunol. Rev..

[3] Misselwitz B., Wyss A., Raselli T., Cerovic V., Sailer A.W., Krupka N., Ruiz F., Pot C., Pabst O. (2020). The oxysterol receptor GPR183 in inflammatory bowel diseases. Br. J. Pharmacol..

[4] Wyss A., Raselli T., Perkins N., Ruiz F., Schmelczer G., Klinke G., Moncsek A., Roth R., Spalinger M.R., Hering L. (2019). The EBI2-oxysterol axis promotes the development of intestinal lymphoid structures and colitis. Mucosal Immunol..

[5] Perucha E., Melchiotti R., Bibby A.J., Wu W., Frederiksen K.S., Roberts C.A., Hall Z., LeFriec G., Robertson K.A., Lavender P. (2019). The cholesterol biosynthesis pathway regulates IL-10 expression in human Th1 cells. Nat. Commun..

[6] Heinig M., Petretto E., Wallace C., Bottolo L., Rotival M., Lu H., Li Y., Sarwar R., Langley S.R., Cardiogenics Consortium (2010). A trans-acting locus regulates an anti-viral expression network and type 1 diabetes risk. Nat. Cell Biol..

[7] Wanke F., Moos S., Croxford A.L., Heinen A.P., Gräf S., Kalt B., Tischner D., Zhang J., Christen I., Bruttger J. (2017). EBI2 Is Highly Expressed in Multiple Sclerosis Lesions and Promotes Early CNS Migration of Encephalitogenic CD4 T Cells. Cell Rep..

[8] Clottu A.S., Mathias A., Sailer A.W., Schluep M., Seebach J.D., Du Pasquier R., Pot C. (2017). EBI2 Expression and Function: Robust in Memory Lymphocytes and Increased by Natalizumab in Multiple Sclerosis. Cell Rep..

[9] Huang J., Lee S.-J., Kang S., Choi M.H., Im D.-S. (2020). 7α,25-Dihydroxycholesterol Suppresses Hepatocellular Steatosis through GPR183/EBI2 in Mouse and Human Hepatocytes. J. Pharmacol. Exp. Ther..

[10] Nevius E., Pinho F., Dhodapkar M., Jin H., Nadrah K., Horowitz M.C., Kikuta J., Ishii M., Pereira J.P. (2015). Oxysterols and EBI2 promote osteoclast precursor migration to bone surfaces and regulate bone mass homeostasis. J. Exp. Med..

[11] Rutkowska A., Preuss I., Gessier F., Sailer A.W., Dev K.K. (2015). EBI2 regulates intracellular signaling and migration in human astrocyte. Glia.

[12] Rutkowska A., Shimshek D.R., Sailer A.W., Dev K.K. (2018). EBI2 regulates pro-inflammatory signalling and cytokine release in astrocytes. Neuropharmacology.

[13] Rutkowska A., O’Sullivan S.A., Christen I., Zhang J., Sailer A.W., Dev K.K. (2016). The EBI2 signalling pathway plays a role in cellular crosstalk between astrocytes and macrophages. Sci. Rep..

[14] Preuss I., Ludwig M.-G., Baumgarten B., Bassilana F., Gessier F., Seuwen K., Sailer A.W. (2014). Transcriptional regulation and functional characterization of the oxysterol/EBI2 system in primary human macrophages. Biochem. Biophys. Res. Commun..

[15] Rosenkilde M.M., Benned-Jensen T., Andersen H., Holst P.J., Kledal T.N., Lüttichau H.R., Larsen J.K., Christensen J.P., Schwartz T.W. (2006). Molecular Pharmacological Phenotyping of EBI2: An orphan sev-en-transmembrane receptor with constitutive activity. J. Biol. Chem..

[16] Norregaard K., Benned-Jensen T., Rosenkilde M.M. (2011). EBI2, GPR18, and GPR17—Three Structurally Related but Biologically Distinct 7TM Receptors. Curr. Top. Med. Chem..

[17] Pereira J.P., Kelly L.M., Xu Y., Cyster J.G. (2009). EBI2 mediates B cell segregation between the outer and centre follicle. Nat. Cell Biol..

[18] Chalmin F., Rochemont V., Lippens C., Clottu A., Sailer A., Merkler D., Hugues S., Pot C. (2015). Oxysterols regulate encephalitogenic CD4+ T cell trafficking during central nervous system autoimmunity. J. Autoimmun..

[19] Hannedouche S., Zhang J., Yi T., Shen W., Nguyen D., Pereira J.P., Guerini D., Baumgarten B.U., Roggo S., Wen B. (2011). Oxysterols direct immune cell migration via EBI2. Nat. Cell Biol..

[20] Liu C., Yang X.V., Wu J., Kuei C., Mani N.S., Zhang L., Yu J., Sutton S.W., Qin N., Banie H. (2011). Oxysterols direct B-cell migration through EBI2. Nat. Cell Biol..

[21] Rutkowska A., Sailer A.W., Dev K.K. (2017). EBI2 receptor regulates myelin development and inhibits LPC-induced demyelination. J. Neuroinflammation.

[22] Crick P.J., Griffiths W.J., Zhang J., Beibel M., Abdel-Khalik J., Kuhle J., Sailer A.W., Wang Y. (2017). Reduced Plasma Levels of 25-Hydroxycholesterol and Increased Cerebrospinal Fluid Levels of Bile Acid Precursors in Multiple Sclerosis Patients. Mol. Neurobiol..

[23] Galiano M., Andrieux A., Deloulme J., Bosc C., Schweitzer A., Job D., Hallak M. (2006). Myelin basic protein functions as a microtubule stabilizing protein in differentiated oligodendrocytes. J. Neurosci. Res..

[24] Olah M., Amor S., Brouwer N., Vinet J., Eggen B., Biber K., Boddeke H.W.G.M. (2011). Identification of a microglia phenotype supportive of remyelination. Glia.

[25] Bauman D.R., Bitmansour A.D., McDonald J.G., Thompson B.M., Liang G., Russell D.W. (2009). 25-Hydroxycholesterol secreted by macrophages in response to Toll-like receptor activation suppresses immunoglobulin A production. Proc. Natl. Acad. Sci. USA.

