# Supplementary material for: EBI2 Is Temporarily Upregulated in MO3.13 Oligodendrocytes during Maturation and Regulates Remyelination in the Organotypic Cerebellar Slice Model
Source: Int J Mol Sci. 2021 Apr 21;22(9):4342. doi: 10.3390/ijms22094342 (PMC8122433; doi:10.3390/ijms22094342)
Supplement: Supplementary file 1 [file ijms-22-04342-s001.zip › ijms-1147722 supp/ijms-1147722.pptx]

## Slide 1
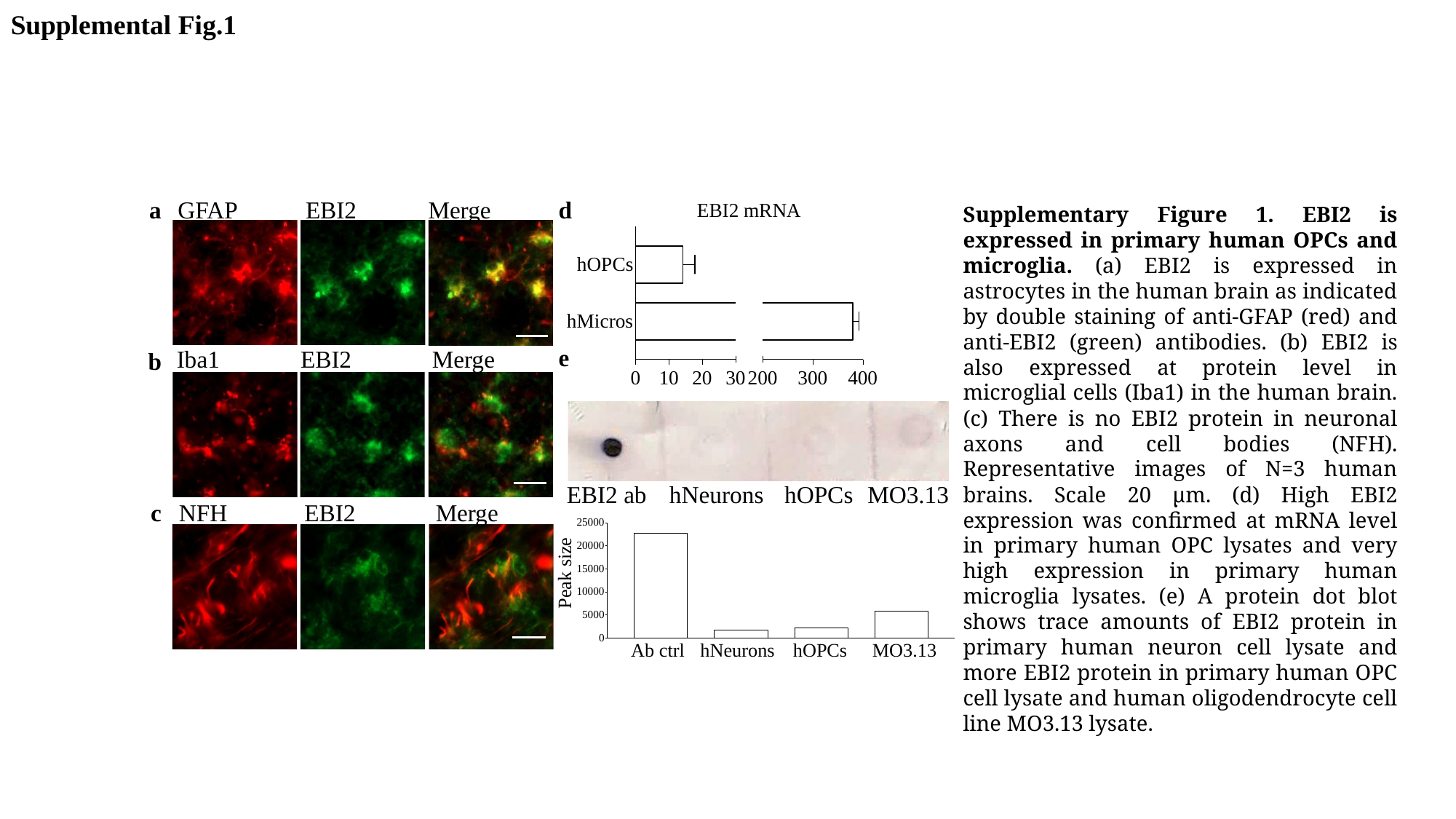

Supplemental Fig.1
a
GFAP
EBI2
Merge
d
Supplementary Figure 1. EBI2 is expressed in primary human OPCs and microglia. (a) EBI2 is expressed in astrocytes in the human brain as indicated by double staining of anti-GFAP (red) and anti-EBI2 (green) antibodies. (b) EBI2 is also expressed at protein level in microglial cells (Iba1) in the human brain. (c) There is no EBI2 protein in neuronal axons and cell bodies (NFH). Representative images of N=3 human brains. Scale 20 μm. (d) High EBI2 expression was confirmed at mRNA level in primary human OPC lysates and very high expression in primary human microglia lysates. (e) A protein dot blot shows trace amounts of EBI2 protein in primary human neuron cell lysate and more EBI2 protein in primary human OPC cell lysate and human oligodendrocyte cell line MO3.13 lysate.
e
Iba1
EBI2
Merge
b
e
EBI2 ab
hNeurons
hOPCs
MO3.13
c
NFH
EBI2
Merge

## Slide 2
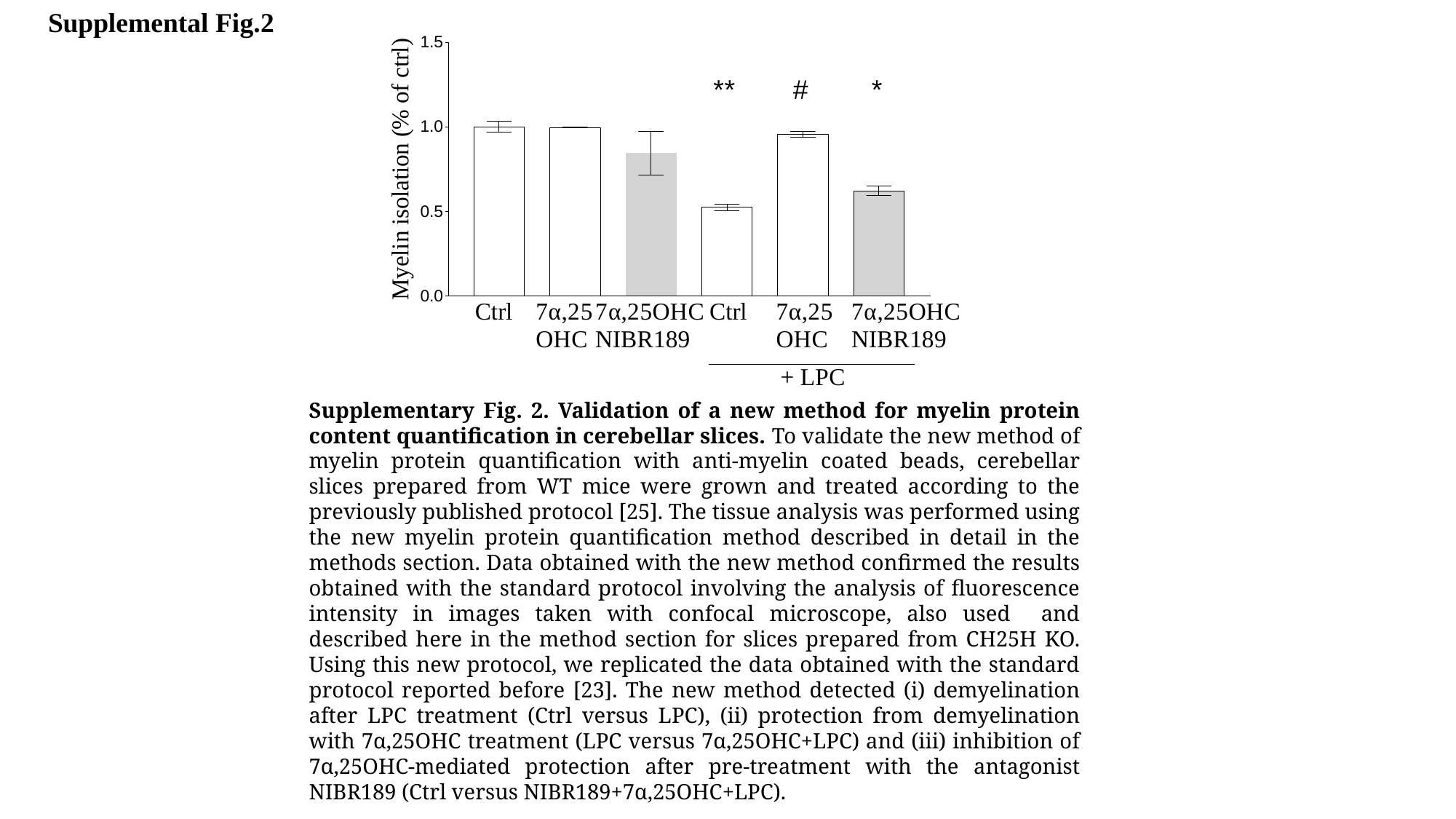

Supplemental Fig.2
Supplementary Fig. 2. Validation of a new method for myelin protein content quantification in cerebellar slices. To validate the new method of myelin protein quantification with anti-myelin coated beads, cerebellar slices prepared from WT mice were grown and treated according to the previously published protocol [25]. The tissue analysis was performed using the new myelin protein quantification method described in detail in the methods section. Data obtained with the new method confirmed the results obtained with the standard protocol involving the analysis of fluorescence intensity in images taken with confocal microscope, also used and described here in the method section for slices prepared from CH25H KO. Using this new protocol, we replicated the data obtained with the standard protocol reported before [23]. The new method detected (i) demyelination after LPC treatment (Ctrl versus LPC), (ii) protection from demyelination with 7α,25OHC treatment (LPC versus 7α,25OHC+LPC) and (iii) inhibition of 7α,25OHC-mediated protection after pre-treatment with the antagonist NIBR189 (Ctrl versus NIBR189+7α,25OHC+LPC).

## Slide 3
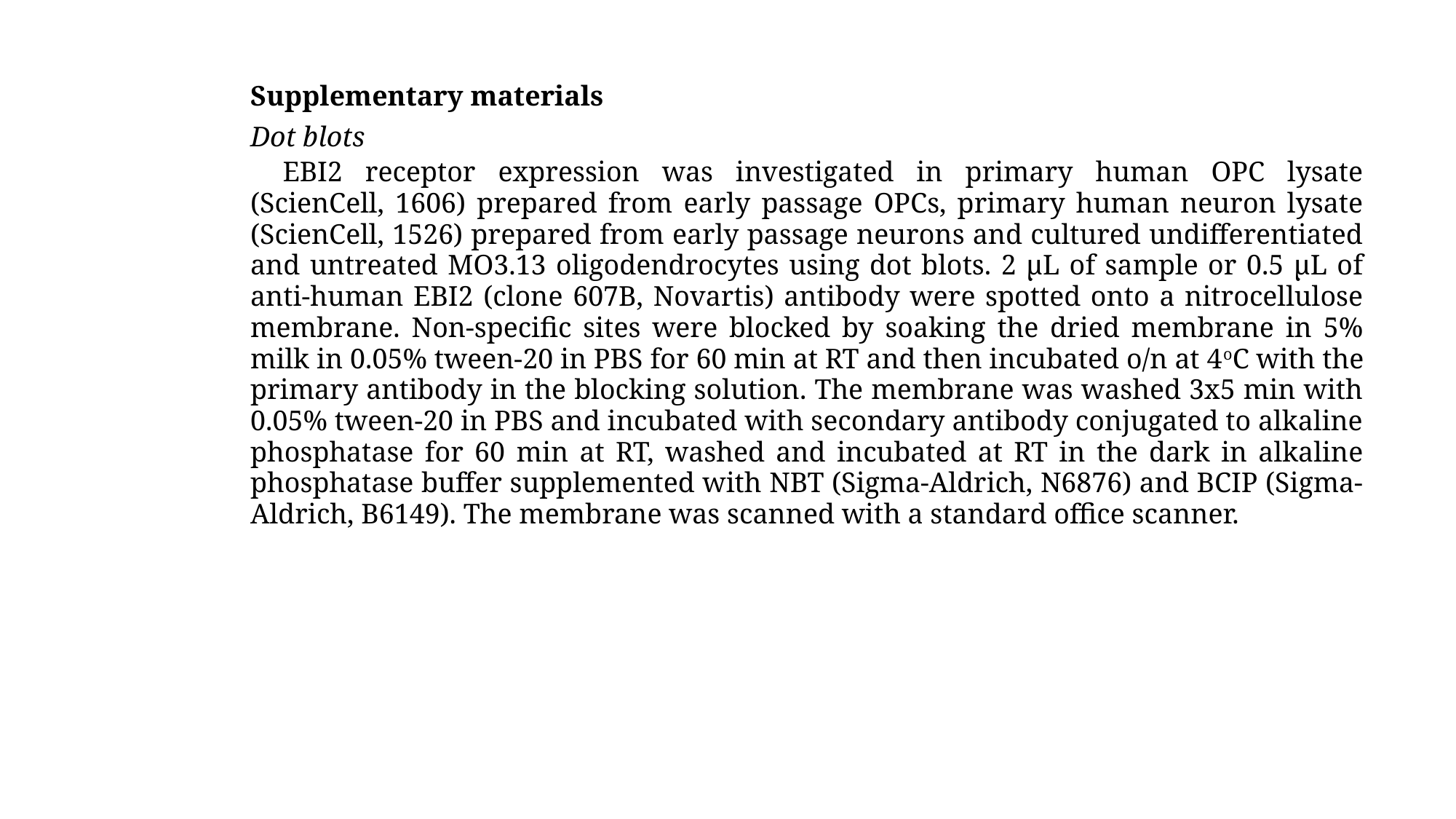

Supplementary materials
Dot blots
EBI2 receptor expression was investigated in primary human OPC lysate (ScienCell, 1606) prepared from early passage OPCs, primary human neuron lysate (ScienCell, 1526) prepared from early passage neurons and cultured undifferentiated and untreated MO3.13 oligodendrocytes using dot blots. 2 μL of sample or 0.5 μL of anti-human EBI2 (clone 607B, Novartis) antibody were spotted onto a nitrocellulose membrane. Non-specific sites were blocked by soaking the dried membrane in 5% milk in 0.05% tween-20 in PBS for 60 min at RT and then incubated o/n at 4oC with the primary antibody in the blocking solution. The membrane was washed 3x5 min with 0.05% tween-20 in PBS and incubated with secondary antibody conjugated to alkaline phosphatase for 60 min at RT, washed and incubated at RT in the dark in alkaline phosphatase buffer supplemented with NBT (Sigma-Aldrich, N6876) and BCIP (Sigma-Aldrich, B6149). The membrane was scanned with a standard office scanner.
